# Nigericin is effective against multidrug resistant gram-positive bacteria, persisters, and biofilms

**DOI:** 10.3389/fcimb.2022.1055929

**Published:** 2022-12-20

**Authors:** Xiaoli Zhu, Anjin Hong, Xihuan Sun, Weijie Wang, Guanghui He, Huan Luo, Zhenhua Wu, Qingyan Xu, Zhiyu Hu, Xiaobing Wu, Donghong Huang, Li Li, Xilin Zhao, Xianming Deng

**Affiliations:** ^1^ State Key Laboratory of Cellular Stress Biology, School of Life Sciences, Faculty of Medicine and Life Sciences, Xiamen University, Xiamen, Fujian, China; ^2^ State-province Joint Engineering Laboratory of Targeted Drugs from Natural Products, Xiamen University, Xiamen, Fujian, China; ^3^ Clinical Microbiology Laboratory, The Second Affiliated Hospital of Fujian Medical University, Quanzhou, Fujian, China; ^4^ State Key Laboratory of Molecular Vaccinology and Molecular Diagnostics, School of Public Health, Xiamen University, Xiamen, Fujian, China

**Keywords:** multidrug-resistant bacteria, persister, biofilm, MRSA, antibiotic, mechanism of action

## Abstract

Multidrug-resistant (MDR) bacteria pose a significant clinical threat to human health, but the development of antibiotics cannot meet the urgent need for effective agents, especially those that can kill persisters and biofilms. Here, we reported that nigericin showed potent bactericidal activity against various clinical MDR Gram-positive bacteria, persisters and biofilms, with low frequencies of resistance development. Moreover, nigericin exhibited favorable *in vivo* efficacy in deep-seated mouse biofilm, murine skin and bloodstream infection models. With *Staphylococcus aureus*, nigericin disrupted ATP production and electron transport chain; cell death was associated with altered membrane structure and permeability. Obtaining nigericin-resistant/tolerant mutants required multiple rounds of challenge, and, cross-resistance to members of several antimicrobial classes was absent, probably due to distinct nigericin action with the GraSR two-component regulatory system. Thus, our work reveals that nigericin is a promising antibiotic candidate for the treatment of chronic or recurrent infections caused by Gram-positive bacteria.

## Introduction

The misuse and overuse of antibiotics has led to the emergence of multidrug-resistant (MDR) bacteria that pose a substantial clinical threat and cause high levels of morbidity and mortality worldwide. Among Gram-positive bacteria, vancomycin-resistant enterococcus (VRE) and methicillin-resistant *Staphylococcus aureus* (MRSA) are the highest ranked in the WHO priority list (Tacconelli et al., 2018). In 2019, more than 100, 000 deaths were attributable to MRSA (Antimicrobial Resistance, 2022). Antibiotic development, especially for infections due to MDR pathogens, has been considered an unmet medical need. Only two new antibiotic classes (lipopeptides and oxazolidinones) have been approved to treat Gram-positive bacteria in the past 20 years (Luepke et al., 2017).

It is important to recognize that the discovery of effective antibiotics against MDR pathogens is very challenging because bacteria have evolved complex and creative strategies to circumvent antibiotic attack. First, bacteria have immense genetic plasticity that results in mutational adaptations, acquisition of genetic material, or alteration of gene expression, all of which can lead to resistance to antibiotics (Lewis, 2013; Munita and Arias, 2016; Hobson et al., 2021). In addition, the generation of ‘persister’ subpopulations can reinitiate growth after antibiotic treatment is terminated, leading to infection recurrence and therapy failure (Harms et al., 2016; Gollan et al., 2019). Particularly problematic is formation of bacterial biofilms that are mainly composed of persisters joined in an extracellular polymeric matrix. Biofilms largely enhance bacterial virulence by allowing bacterial cells to effectively adhere to solid surfaces and protecting the cells against host immune molecules and antibiotics (Bridier et al., 2011; Hancock et al., 2021). There is an urgent need to develop effective antibiotics or drug combinations to combat MDR pathogens, especially those that can kill persister cells and biofilms. In addition, they should have mechanisms of action (MoA) distinct from existing drugs to avoid cross-resistance. These criteria are difficult to satisfy.

Through continuous efforts to search for antibiotics that can effectively treat MDR pathogens, we found a polyether ionophore nigericin (NIG) has potent bactericidal ability against a variety of clinical MDR Gram-positive bacteria, including MRSA and VRE. NIG has been shown to have a broad spectrum of biological activities since 1960 (Kevin Ii et al., 2009), including antibacterial activities against *S. aureus*, *Bacillus cereus*, *Enterococcus faecalis* and mycobacterial species (Harold and Baarda, 1969; Wu et al., 2009; Huang et al., 2017). However, the antibacterial activity of NIG has not been systemically investigated. Most studies simply measured minimal inhibitory concentration (MIC) with one or a few bacterial strains (Wu et al., 2009; Taechowisan et al., 2013; Huang et al., 2017; Kumarsahu et al., 2020). No study with a large panel of clinical isolates, especially those with MDR profiles, has been carried out. The *in vivo* efficacy of NIG has not been investigated before. In addition, although NIG was found to be capable of transporting cations across biological membranes as early as several decades ago (Harold, 1969), the bactericidal mechanism of NIG has not been further elucidated since then. Therefore, we performed systematic investigation of the antibacterial activity, *in vivo* efficacy and mechanisms of antimicrobial action for NIG.

We found that NIG has potent bactericidal ability against a variety of clinical MDR Gram-positive bacteria, including MRSA and VRE. Moreover, NIG retains antimicrobial activity against persisters, reduces biofilms and exhibits a low propensity for resistance development. NIG also exhibits considerable *in vivo* efficacy in a deep-seated mouse biofilm infection model, a chronic trauma infection model, and an acute murine bloodstream infection model. Further mechanistic studies revealed that NIG disrupts cell membrane, leading to increases in permeability that account for the lethal effects of the compound. Moreover, the decrease in the positive charge on the cell membrane by depletion of the GraSR two-component regulatory system leads to NIG resistance. The profound antimicrobial activity and the distinct MoA of NIG from those of other known classes of antibiotics make it a promising antibiotic candidate for the treatment of chronic or recurrent infections caused by MDR Gram-positive bacteria.

## Materials and methods

### Bacterial strains and culture media

Clinical isolates including *S. aureus*, MRSA, *Staphylococcus epidermidis*, *Enterococcus faecium*, *Enterococcus faecalis*, *Streptococcus pneumoniae* and *Streptococcus agalactiae* were obtained from Zhongshan Hospital of Xiamen University and The Second Affiliated Hospital of Fujian Medical University. Each strain was grown from a single colony and cultured in Brain Heart Infusion (BHI) broth (Solarbio, Bejing, China) at 37°C with 220 rpm shaking overnight with the following exceptions: RN450 was cultured in Mueller-Hinton (MH) broth (OXOID, UK), and Muller- Hinton2 (MH2) broth (OXOID, UK) was used to culture RN450 persister cells. 1.5% agar (BD, USA) was added to broth media to obtain solid media. Sheep blood (10%) (Yuanye, Shanghai, China) was added to growth medium for *S. pneumoniae* and incubation was at 37°C in the presence 5% CO_2_.

### Measurement of minimum inhibitory concentration

MIC was detected by the broth microdilution method based on the Clinical and Laboratory Standards Institute (CLSI) guideline (CLSI, 2021). The MIC was determined as the lowest concentration of the drug that blocked visible bacterial growth (no turbidity increase compared with no bacteria negative control and no drug positive control) after an overnight incubation. MIC_50_ was determined as MIC at which at least 50% of the strains were inhibited, while the MIC_90_ inhibits 90% of the strains tested.

### Time-kill assays

Time-killing experiments were performed as described previously by Smith and Romesberg (Smith and Romesberg, 2012). Overnight cultures of strains were diluted 1:100 into MH liquid medium. Killing kinetics was measured by incubating exponentially growing liquid cultures (~1 × 10^8^ CFU/mL) with drugs at the indicated concentration for the indicated times after which samples were taken, serially diluted, and plated on drug-free agar in 3 × 10 μL technical repeats. Agar plates were then incubated at 37°C for 24 h to determine bacterial counts. A detection limit of 33 CFU/mL was achieved since the assay can detect no less than 1 colony in 3 × 10 μL serially diluted samples (Le et al., 2020).

### Checkerboard assay

After determining the individual MICs of each antibiotic, overnight culture of *S. aureus* RN450 was diluted 1:100 in fresh MH medium and grown at 37°C, 220 rpm until the OD_600_ reached 0.25. Then RN450 culture was diluted 1:5000 in fresh MH and distributed in 100 uL aliquots into 96-well plates (Fisher Scientific, US). Serial dilutions of nigericin and another antibiotic, ranging from 1/32 × MIC to 4 × MIC were added to wells. Fractional inhibitory concentration index (FICI) was calculated using the formula: FIC = FIC_A_ + FIC_B_ = MIC_A+B_/MIC_A_ alone +MIC_B+ A_/MIC_B_ alone. The FIC index was interpreted as follows: ≤ 0.5 synergy, 0.5-0.75 partial synergy, >0.75-1 additive, >1-4 no effect, and >4 antagonism. Three independent experiments were performed for each antimicrobial tested

### Persister cell assays

The persister cell assay I was a modification of the method of P. Le et al. (Le et al., 2020). To obtain persister cells, an overnight culture of *S. aureus* was diluted 1:100 into fresh tryptic soy broth (TSB, 50 mL in 250 mL culture flasks) and distributed in 100 μL aliquots to 96-well plates (Fisher Scientific, US). They were then grown at 37°C, 220 rpm for 16 h. Cell numbers were determined by plating following serial dilution. Persisters were prepared by 24 h treatment with ciprofloxacin (78 μM, 100 × MIC) at 37°C, 220 rpm. Persisters were washed with PBS containing 1% MH2. Nigericin (1 μg/mL, 8 × MIC; 2 μg/mL, 16 × MIC) and rifampicin (0.1 μM, 10 × MIC) were added, followed by incubation at 37°C, 220 rpm for 196 h. At various times, samples were withdrawn, serially diluted, and plated for determination of CFU/mL.

Persister cell assay II, which is strictly a stationary phase cell killing assay (because stationary cells are considered as persisters in previous work (Springer et al., 2016) due to their ability to tolerate antimicrobial killing), was performed as follows. A culture of *S. aureus* 25923 was grown (37°C, 200 rpm) in TSB medium from an overnight culture for 20 h to achieve stationary phase. Serial dilutions were prepared and plated on agar plates to determine cell number for the inoculum. 1 mL portions were aliquoted and treated (37°C, 200 rpm) with 0.1% EtOH, tigecycline (2 μg/mL, 16 × MIC), or nigericin (1 μg/mL, 8 × MIC) for various times at which samples were taken, harvested by centrifugation (10,000 × g, 3 min), washed three times with PBS, serially diluted, and plated on agar for determination of CFU/mL.

### Minimum biofilm eradication concentration

Minimum biofilm eradication concentration (MBEC), which is a modification of the procedure of *Le et al.* (Le et al., 2020), was defined as the lowest antimicrobial concentration that caused a reduction of > 60% (e.g. MBEC_60_) of biofilm biomass based on the crystal violet release assay described below. To obtain biofilm, an overnight culture of *S. aureas* RN450 was diluted 1:100 in fresh TSB medium and 200 μL aliquots were placed in 96-well plates (Fisher Scientific, USA) and grown at 37°C incubation for 24 h in non-shaking conditions after which the supernatant was removed from the wells. Various concentrations of nigericin were diluted in TSB medium and then added to the plate with the established biofilm for incubation at 37 °C for 96 h. The wells in the plate were washed with PBS and then dried at 37°C for 3 h and then at room temperature overnight. The biofilm was stained by crystal violet and washed with PBS. 95% ethanol was then added to each well to dissolve the remaining colorant. The OD_595_ was measured with a microplate reader (Thermo Fisher Scientific, US). Data were obtained from three independent experiments.

### Assays for emergence of resistance

Resistance development kinetics were measured as described in Le et al. (Le et al., 2020). For resistance development by sequential passage, exponentially growing *S. aureus* RN450 was diluted 1:100 into MH medium containing various concentrations of nigericin, vancomycin, or ofloxacin. Bacteria were incubated at 37°C with shaking at 200 rpm, and passaged at 24 h intervals in the presence of 0.25, 0.5, 1, 2, 4 × MIC nigericin, vancomycin, or ofloxacin. Cultures from the second highest concentration that allowed bacterial growth were diluted 1:100 into fresh media containing various concentrations of each antimicrobial (0.25, 0.5, 1, 2, 4 × MIC) for next round challenge. This serial passaging was repeated daily for 32 days. The MIC to each challenge drug was measured after each round of challenge and plotted as a function of challenge time (round of challenge).

### Propydium iodide staining

*S. aureus* RN450 was grown at 37°C, with shaking at 220 rpm for 2 h after diluting an overnight culture by 100-fold. Nigericin (1 μg/mL, 8 × MIC) was added, and RN450 was incubated for 7 h. The bacteria were stained using a PI Staining Kit (Sangon Biotech, China). The image was obtained by LSM 780 Laser Confocal Microscope (Zeiss, Germany). Data were obtained from three independent experiments.

### Measurement of membrane potential

RN450 was grown in MH medium to OD_600_ = 0.25. Culture (100 μL) was distributed to wells in 96-well plates (Fisher Scientific, USA). After 3 min of measuring background fluorescence, the culture was treated with DiSC3(5) (Sigma Aldrich) (1 μM final concentration), and fluorescence was measured for another 7 min. Then 0.0625 μg/mL nigericin (0.5 × MIC), 0.5 μg/mL daptomycin (0.5 × MIC), or EtOH as a solvent control, was added respectively. Fluorescence was recorded for 1 h at an excitation wavelength of 610 nm and an emission wavelength of 660 nm. Data were obtained from three independent experiments.

### Scanning electron microscopy and transmission electron microscopy

RN450 was grown in MH from overnight cultures following a 1:100 dilution at 37°C, 220 rpm for 2 h. Ampicillin (6 × MIC, 9 h), nigericin (4 × MIC, 6 h) and 0.1% EtOH were added and incubated. Samples were washed with PBS (Thermo Fisher Scientific, US) three times and fixed by 2.5% glutaraldehyde overnight at 4°C. For SEM, the samples were dehydrated by incubation with 30%, 50%, 70%, 80%, 95% and 100% gradient concentrations of ethanol. The fixed samples were spray-coated with a thin layer of gold and examined by scanning electron microscopy SUPRA55 SAPPHIRE (Carl Zeiss AG, German).

For TEM, the fixative was removed by centrifuging, and then samples were embedded in agar (BD, USA), followed by fixation using 2.5% glutaraldehyde at 5°C overnight. After washing with PBS three times at 4°C (each wash involving a 15 min incubation), samples were cut into ultrathin sections (70 nm) with a diamond knife. Images were recorded digitally with a transmission electron microscope HT-7800 (Hitachi, Tokyo, JP).

### Enrichment of nigericin-resistant *S. aureus*

*S. aureus* strain RN450 was grown from 100-fold dilution of an overnight culture at 37°C, 220 rpm for 2 h and then incubated with nigericin (2 μg/mL, 16 × MIC) for 18 h. The cells were then collected by centrifuging and washed three times with fresh MH, and then cultured in fresh MH. The culture was treated with nigericin for 18 h and then the drug was removed by centrifugation. After 6 rounds, the culture was diluted and applied to an agar plate containing 0.5 μg/mL nigericin. Colonies that grew on the plate were cultured in fresh MH, and the MIC was determined. Isolates that maintained MIC values 4-fold greater than the parental RN450 strain after passage growth on drug-free agar for five rounds were considered having stable reduced susceptibility to nigericin.

### Allelic replacement in *S. aureus* RN450

Gene editing of strain RN450 was based on the procedure by T. Bae and O. Schneewind with modification using allelic replacement plasmid pKOR1 (Bae and Schneewind, 2006). Plasmid transformation of RN450 and growth at 43°C were used to select for homologous recombination and pKOR1 integration into the bacterial chromosome. Anhydrotetracycline was used to select for chromosomal excision and loss of plasmid by inducting *secY* antisense transcripts *via* the Pxyl/tetO promoter.

### Iodonitrotetrazolium chloride reduction assay

*S. aureus* RN450 cells were grown from an overnight culture to exponential phase (37°C, shaking at 200 rpm), and they were then treated with 1 μg/mL daptomycin, 0.125 μg/mL nigericin, or EtOH for 1 h. Cells were then harvested by centrifugation (10,000 × g, 3 min), resuspended in PBS, and kept on ice. Iodonitrotetrazolium chloride (INT, Acmec, cat.# I32350) was added to bacterial suspension (1 × 10^7^ CFU/mL) to achieve a final concentration of 3 mM in a total volume of 3 mL. Tubes were mixed vigorously, and absorbance at 490 nm was determined at 10-min intervals. Tubes were incubated at 30°C between measurements.

### Detection of ATP levels

ATP levels were determined by adaptation of the method of Patton et al. *S. aureus* RN450 cells were grown from an overnight culture to exponential phase (37°C, shaking at 200 rpm). Cultures were treated with 1 μg/mL daptomycin, 0.125 μg/mL nigericin, or EtOH for 1 h. After treatment, cells were concentrated by centrifugation and resuspended in PBS. ATP concentrations were determined using an ATP bioluminescent assay kit (Beyotime, cat.#S0026B, Shanghai, China) according to the manufacturer’s instructions. Bioluminescence was measured by a Thermo Fisher Scientific Varioskan Flash Multiplate Reader.

### Reverse transcription and quantitative real-time PCR

Total RNA was extracted from *S. aureus* RN450 and *graS* ^FS^ strains using Total RNA Isolation Kit V2 (Vazyme, Nanjing, Jiangsu, China, cat.# RC112-01) according to the manufacturer’s instructions. Reverse transcription was performed with Hifair^®^ III 1st Strand cDNA Synthesis SuperMix (Yeasen, Shanghai, China, cat.# 11141ES10). Quantitative PCR was performed with SYBR green master mix (Yeasen, Shanghai, China, cat.# 11201ES03). Standard curve was generated by serially diluting the pOS1-*mprF* or pOS1- *dlt* plasmids (10^1^-10^8^ copies) and determining the corresponding cycle threshold. According to the standard curves, the mRNA copies of each sample were calculated and normalized to internal control (16S rRNA) of each *S. aureus* sample before mRNA abundance from different strains treated under the same condition or from the same stain undergoing different treatment was compared.

### Animal studies

ICR mice were purchased from the Laboratory Animal Center of Xiamen University. Animal studies were approved and performed according to the guidelines of the Xiamen University Laboratory Animal Center. Female 6- to 8- week-old ICR mice were used in all experiments. The protocol (approval # XMULAC20190028) was approved by the institutional animal care and use committee of the Laboratory Animal Center of Xiamen University.

### Deep-seated mouse biofilm infection model

A previously reported deep-seated mouse biofilm infection model (Conlon et al., 2013) was used with modifications. 100 μL of stationary-phase *S. aureus* USA300 (5 × 10^7^ CFU) was injected to the thigh of each mouse. Starting at 24 h post-infection, mice were administrated with 200 µL of nigericin (1 mg/kg, dissolved in 20% (w/v) Kolliphor^®^ HS 15 (Sigma-Aldrich, USA, cat.#42966)), vancomycin (50 mg/kg), or vehicle intraperitoneally every 12 h for 3 days. Mice were sacrificed 4 days post-infection, and their infected thighs were homogenized, diluted, and plated on MH agar for determination of CFU.

### Murine wound infection

Murine wounds were generated as round lesions having a diameter of 1 cm on the back of each mouse. Each wound was then infected with 10 μL of 3 × 10^7^ CFU/mL *S. aureus* USA300. Staring at 24 h post-infection, nigericin (0.025 μg per wound) or mupirocin (200 μg per wound), diluted in 0.1% EtOH, was applied to the wounds once daily for 9 days. The wounds were monitored at 1, 5- and 10-days post-infection. The wound tissues were excised and grounded, and aliquots of the grounded tissue were diluted in normal saline and plated on drug-free agar to determine CFU. A portion of the wound tissue was also subjected to hematoxylin and eosin (H&E) staining for histopathological analysis.

### Murine bloodstream infection model

Mice were infected by intravenous injection of 100 μL 1 × 10^8^ CFU/mL *S. aureus* USA300. Starting at 4 h post-infection, nigericin (0.5, 1 mg/kg, dissolved in 20% (w/v) Kolliphor^®^ HS 15), vancomycin (5 mg/kg), or vehicle (20% (w/v) Kolliphor^®^ HS 15) in a volume of 200 µL was administrated *via* the intraperitoneal route every 12 h for 3 d. Survival curves were recorded during 3 days of treatment. For determining the bacterial loads of USA300, the mice were sacrificed 3 days post-infection, and their hearts, livers, spleens, lungs, and kidneys were homogenized, diluted, and plated on MH agar for determination of CFU.

## Results

### NIG is a broad-spectrum, bactericidal antibiotic against MDR gram-positive bacteria

**Figure 1:**
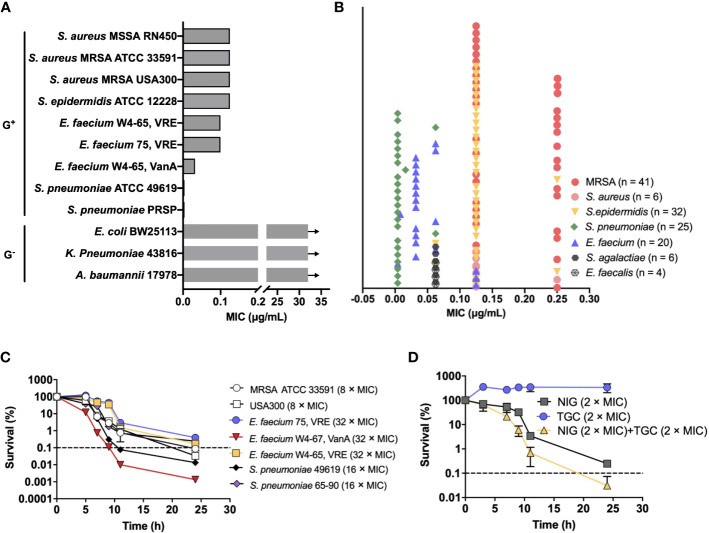
Nigericin activity against drug-resistant bacteria. **(A)** Bacteriostatic activity with selected representative bacterial species. Nigericin MIC is shown for the listed strains of Gram-positive and Gram-negative bacteria. Arrows indicate that the MIC exceeded the highest nigericin concentration tested. **(B)** Bacteriostatic activity with a collection of 134 drug-resistant clinical isolates. **(C)** Bactericidal activity. Exponentially growing bacterial cultures were treated with the indicated (after species name in parenthesis) concentrations of nigericin for the indicated times after which percent survival was plotted as a function of treatment time. **(D)** Synergistic bactericidal activity between nigericin and tigecycline. Exponentially growing *S. aureus* strain RN450 cultures were treated with the indicated concentrations of Nigericin (NIG), tigecycline (TGC), or the two in combination for the indicated times. **(A, B)** Similar results were obtained from 3 replicate assays. **(C, D)** Dashed line indicates 0.1% survival. Data are average of 3 biological replicates; error bars indicate SD.

To identify potential agents that could effectively treat MDR pathogens, we screened an in-house natural product library (~4,500 compounds) using the MRSA USA300 strain. The compounds with a minimal inhibitory concentration (MIC) lower than 8 μg/mL were further applied to sensitivity detection among a panel of the highest ranked Gram-positive bacteria in the WHO priority list, including methicillin-resistant *Staphylococcus aureus* (MRSA), vancomycin (VAN)-resistant enterococcus, and penicillin-resistant *Streptococcus pneumoniae* (PRSP). NIG attracted our attention since it exhibited potent activity, with MICs ranging from 0.004-0.125 μg/mL, which were lower than those of commonly used antimicrobials such as VAN, ampicillin, and penicillin (**Figure 1A**, **Supplementary Table S1**). In contrast, NIG was inactive against representative Gram-negative bacterial strains, such as *Escherichia coli* BW25113, *Acinetobacter baumannii* 17978, and *Klebsiella pneumoniae* 43816 (e.g. MIC > 32 μg/mL, **Figure 1A**, **Supplementary Table S1**).

To assess the spectrum of anti-Gram-positive bacteria activity, we further determined the MICs of NIG for a collection of 134 clinical MDR strains consisting of *S. aureus* (n = 47), *Staphylococcus epidermidis* (n = 32), *Enterococcus faecalis* (n = 4), *E. faecium* (n = 20), *S. pneumoniae* (n = 25), and *Streptococcus agalactiae* (n = 6). Most of these MDR strains are significantly resistant to β-lactam antibiotics and that more than half of them are resistant to aminoglycosides and fluoroquinolones (**Supplementary Table S2**). Encouragingly, NIG exhibited potent activity against these clinical MDR strains, with MIC values ranging from 0.004-0.25 μg/mL (**Figure 1B**). The MIC_90_ values of NIG were 4-160-fold lower than those of frequently used clinical antibiotics, such as oxacillin (OXA), rifampicin (RIF), and moxifloxacin (MXF), and even the most efficacious VAN (**Supplementary Table S2**). Thus, low MIC with a wide variety of clinical isolates indicates potential use of NIG against MDR Gram-positive pathogens.

We next measured bactericidal action of NIG. Results showed that NIG exhibited bactericidal activities against MRSA 33591 and USA300, vancomycin-resistant *E. faecium*, and penicillin-resistant *S. pneumoniae* (**Figure 1C**). Bactericidal activity depended on incubation time, not drug concentration over the range tested (**Supplementary Figure 1**), indicating that NIG is a time-dependent killing antimicrobial.

We then performed a checkerboard assay to determine the synergistic bacteriostatic effect of NIG in combination with other antibiotics. The results showed that NIG potentially synergized with tigecycline (TGC), OXA, and MXF, as their fractional inhibitory concentration (FIC) index was at or below 0.5 (**Supplementary Table S3**). Time-kill curves showed that a combination of 2 × MIC NIG plus TGC at 2 × MIC, a sub-lethal concentration for TGC itself, reduced viable bacteria 10-fold more than NIG alone during a 24-h treatment (**Figure 1D**), while the combination of NIG with sublethal concentrations of OXA or MXF had no synergistic lethal effect (**Supplementary Figure S2**). Taken together, these data suggest the bactericidal potency synergistic effect of NIG with other antimicrobials against MDR Gram-positive bacteria.

### NIG largely reduces the viability of persisters, stationary phase cells, and established biofilms

**Figure 2:**
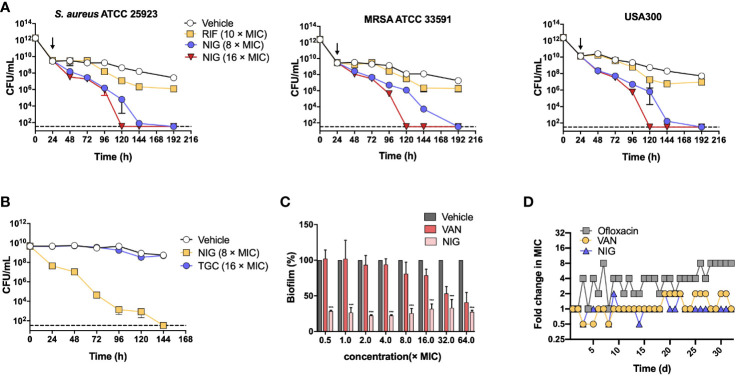
Nigericin treatment eliminates *S. aureus* persisters, stationary phase cells, and biofilms. **(A)** Killing of persisters. Overnight cultures of 3 *S. aureus* strains were treated with 100 × MIC of ciprofloxacin for 24 h to enrich persisters. Ciprofloxacin was removed and the indicated concentrations of nigericin (NIG) or rifampicin (RIF) were added (downward arrow) to kill the enriched persister subpopulations for the indicated times. Viable count was recorded and plotted as a function of treatment time. Vehicle (0.1% EtOH) indicating solvent for the drug was added as a control. Dash line indicates detection limit. Data are average of 3 biological replicates; error bars indicate SD. **(B)** Comparison of nigericin and tigecycline for killing of persisters. Persisters of *S. aureus* ATCC 25923 were generated from an overnight culture for 20 h to achieve stationary phase, and were then treated with nigericin (NIG, 8 × MIC), tigecycline (TGC, 16 × MIC) or Vehicle (0.1% EtOH) for the indicated times. Data were then processed as in panel **(A)**. **(C)** Killing of biofilms. *S. aureus* ATCC 25923 biofilms were treated for 24 h with the indicted concentrations of nigericin (NIG), vancomycin (VAN), or Vehicle (0.1% EtOH). After treatment, biofilms were washed and then dispersed by PBS. Samples were then serially diluted and plated on agar for viable count determination. Percent survival was calculated, using vehicle as a control, and plotted as a function of drug concentration. Data are average of 3 biological replicates; error bars indicate SD. *** p<0.001. **(D)** Emergence of drug resistance. *S. aureus* RN450 cultures were treated with 0.25, 0.5, 1, 2, 4 × MIC nigericin (NIG), vancomycin (VAN), or ofloxacin (OFL) with daily serial passaging. At each passage samples were taken for MIC determination and the MIC values determined were ploted as a function of passage numbers (treatment times). Similar results were obtained from 3 replicate assays.

We next evaluated the ability of NIG to kill *S. aureus* persister cells using *S. aureus* 25923, MRSA 33591, and MRSA USA300 strains. Persister cells were generated from individual stationary strain cultures by ciprofloxacin treatment (100 × MIC, 24 h), which killed >99% of the bulk population. The number of persister cells was effectively reduced upon NIG treatment in a time- and concentration-dependent manner. NIG at 16 × MIC completely eradicated *S. aureus* persistent bacteria within 120 h to below the detection limit (33 CFU/mL, **Figure 2A**). In contrast, RIF showed a weak effect on the viability of MDR *S. aureus* persister cells (**Figure 2A**), confirming previous work (Conlon et al., 2013; Guo et al., 2018; Le et al., 2020). Using cells produced *via* another persister generation procedure (Springer et al., 2016), we further confirmed that NIG displayed significant bactericidal ability against *S. aureus* 25923 persister (stationary phase) cells, since 100% of the cells were killed at 144 h with 8 × MIC (**Figure 2B**). Staphylococcal biofilms, mainly composed of persisters (Waters et al., 2016), could also be effectively reduced by >60% with 0.0625 μg/mL (0.5 × MIC) NIG treatment (MBEC_60_ = 0.0625 μg/mL), whereas VAN had little effect on survival of biofilm bacteria up to 16 × MIC. Increasing concentrations of NIG did not display additional killing effects (**Figure 2C**), as expected from its time-dependent killing property with planktonic cells.

The potential for *S. aureus* RN450 to develop resistance to NIG was then investigated. Upon serial passage for a period of 32 days, no NIG-resistant mutants emerged, and the population MIC remained constant for NIG, as it did for VAN after a similar passage procedure. In contrast, the MIC for the fluoroquinolone ofloxacin gradually increased throughout a similar passage experiment (**Figure 2D**), as expected from earlier work with quinolones and Gram-negative bacteria (Malik et al., 2010; Malik et al., 2012). Overall, NIG displays bactericidal properties in killing persister cells and eradicating biofilms, and exhibits a low propensity for resistance development, which is superior to compounds commonly used to treat these pathogens.

### NIG is efficacious in mouse models of USA300 infection

**Figure 3:**
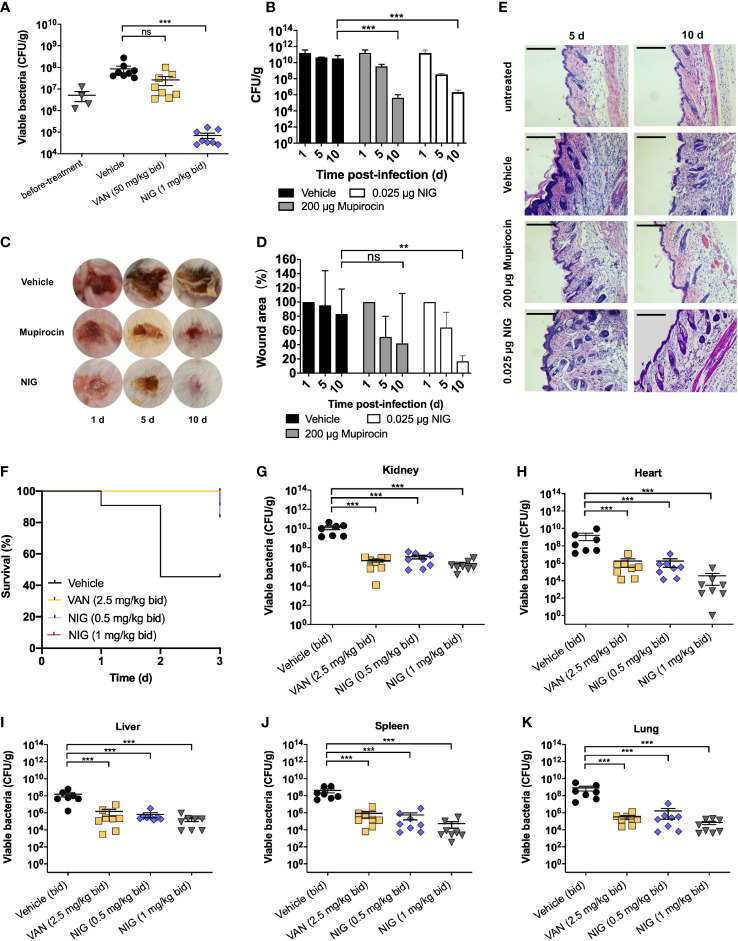
Nigericin efficacy for treatment of *S. aureus* infection. **(A)** Deep-seated mouse biofilm infection model using *S. aureus* strain USA300 (n = 8). After acclimation, mice were subjected to thigh injection of 5 × 10^7^ CFU of stationary-phase *S. aureus* USA300. Starting 24 h after infection, mice were treated twice daily with vancomycin (VAN, 50 mg/kg), nigericin (NIG, 1 mg/kg), or vehicle (20% (w/v) Kolliphor^®^ HS 15) for 3 days. Bacterial burden in thigh was determined after 3 days of treatment. **(B–E)** Trauma infection model using *S. aureus* strain USA300 (n = 5). After acclimation, mice were subjected to subcutaneous wound generation followed by infection (3 × 10^7^ CFU/wound) of *S. aureus* strain USA300. Starting 24 h post-infection, topical daily treatment with nigericin (0.025 μg per wound), mupirocin (200 μg per wound), or a vehicle control was carried out for 9 days. Samples were taken at days 1, 5, and 10 for analysis presented in panels **(B–E)**. **(B)** Wound viable bacterial count. Wound tissues were dissected, homogenized, serially diluted, and plated on agar for enumeration of viable bacteria. **(C)** Visualization of wounds at indicated times after initiation of treatment. **(D)** Wound areas were measured. **(E)** Wound histopathology was recorded. Skin portions were harvested, sectioned, and stained with H&E. They were then examined by light microscopy. Scale bar, 250 μm. **(F-K)** Murine blood infection using *S. aureus* USA300. After acclimation, mice were subjected to tail vein injection of 1 × 10^7^ CFU of *S. aureus* USA300. Starting 4 h after infection, mice were treated twice daily with vancomycin (VAN, 5 mg/kg), nigericin (NIG, 0.5 mg/kg/1 mg/kg), or vehicle (20% (w/v) Kolliphor^®^ HS 15) for 3 days. Animal survival was monitored daily and presented in **(F)**. Animals in the vehicle (control) arm were so sick at day 3 that they were subjected to euthanasia. Bacterial burden in various organs was determined after 3 days of treatment and presented in panels **(G)** (Kidney), **(H)** (Heart), **(I)** (Liver), **(J)** (Spleen), and **(K)** (Lung). Each point represents an individual mouse. Horizontal lines represent the mean ± SEM. n = 7 for vehicle group, n = 8 for vancomycin (VAN) and nigericin (NIG) groups. ns, not significant; **p < 0.01; ***p < 0.001.

Since the outstanding antibacterial properties of NIG and its efficacy has not been evaluated in animal models of infection, we further tested the *in vivo* efficacy of NIG using mouse models of *S. aureus* USA300 infection. We first used deep-seated mouse biofilm infection model with the *S. aureus* USA300 strain to mimic human deep-seated chronic infections. 5 × 10^7^ CFU of *S. aureus* USA300 was injected to the thigh of each mouse. Twenty-four hours after bacterial infection, mice were administrated intraperitoneally with 50 mg/kg VAN or 1 mg/kg NIG every 12 hours for 3 days. VAN treatment did not significantly reduce *S. aureus* USA300 abundance, which suggests the presence of a VAN-tolerant bacterial population in this model (**Figure 3A**). Remarkably, treatment with NIG led to an approximately 10^3^-fold reduction of USA300 load in the infected thigh (**Figure 3A**). The potency of NIG reducing the stationary cells and biofilm populations *in vivo* was encouraging.

Then, we used a trauma infection model with the *S. aureus* USA300 strain. Wounds were punched on the back skin of mice, followed by infection with *S. aureus* USA300. One day after infection, NIG ointment was applied once daily for 9 days. The results showed that NIG reduced USA300 loads approximately 10^4^-fold in the wound (**Figure 3B**). Meanwhile, NIG treatment reduced the wound size by 80% after 10 days, as did mupirocin, an established anti-staphylococcal agent (**Figures 3C, D**), indicating that NIG, as well as mupirocin, may promote wound healing by drastically reducing bacterial load at the site of infection. Through hematoxylin and eosin (H&E) staining of the infected skin, disruption of the skin epidermis layer and infiltration of numerous lymphocytes in the interstitium were observed in the USA300-infected mice, while the mice treated with mupirocin or NIG showed more healed skin structures and decreased lymphocyte levels in the interstitium (**Figure 3E**). Notably, the efficacy of NIG was comparable to that of mupirocin but at an 8,000-fold lower concentration.

A murine model of bloodstream infection was further used. One hour after injection with a lethal dose of USA300 through the tail vein, mice were treated with drugs (via ip) every 12 h for 3 days. Treatment with 1 mg/kg NIG significantly prolonged the survival of USA300-infected mice, which was comparable to that of mice treated with 5 mg/kg VAN (**Figure 3F**). The bacterial loads of USA300 in the heart, liver, spleen, lung and kidney were also detected at 72 h post-infection. NIG treatment led to a 1,000- to 10,000-fold reduction in the bacterial burden in the major organs. The effect of 1 mg/kg NIG was slightly better than that of 2.5 mg/kg VAN (**Figures 3G–K**). These results demonstrate the *in vivo* efficacy of NIG in the treatment of *S. aureus* infection.

### NIG disrupts membrane structure and the permeability barrier

**Figure 4:**
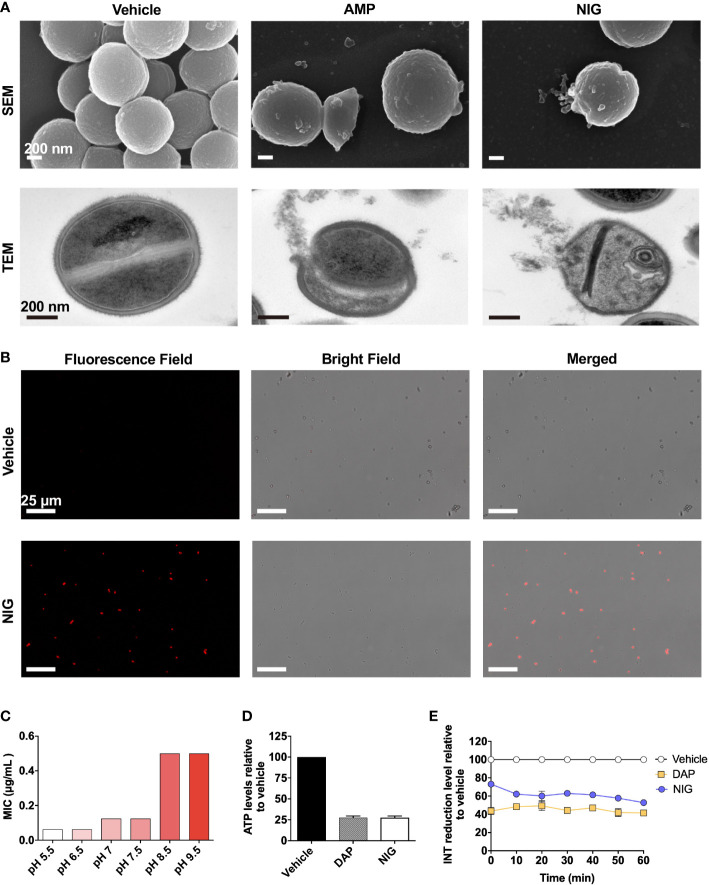
Nigericin disrupts membrane integrity. **(A)** Electron microscopy. SEM and TEM showing *S. aureus* RN450 upon treatment with nigericin (NIG; 4 × MIC, 6 h), ampicillin (AMP; 6 × MIC, 9 h) or vehicle (0.1% EtOH, 6 h). Images are representative of 208 recordings from three independent experiments. Scale bar, 200 nm. **(B)** Propidium iodide staining. *S. aureus* RN450 cultures were incubated with vehicle (0.1% EtOH) or nigericin (NIG; 8 × MIC) for 7 h and then stained with propidium iodide, and observed with a fluorescence microscope. Scale bar, 25 μm. **(C)** Effect of extracellular pH on nigericin MIC with *S. aureus* RN450. **(D)** Effect of nigericin on intracellular ATP levels. *S. aureus* RN450 was treated with daptomycin (DAP, 1 × MIC), nigericin (NIG, 1 × MIC), or vehicle (0.1% EtOH) for 1 h. **(E)** Effect of nigericin on relative reduction of the electron transport chain. *S. aureus* strain RN450 was treated with daptomycin (DAP, 1 × MIC), nigericin (NIG, 1 × MIC), or 0.1% EtOH (vehicle) for the indicated times. **(D-E)** Data are average of 3 biological replicates; error bars indicate SD.

NIG is an ionophore that carries K^+^ and H^+^ through the membrane (Cook et al., 2014). To gain additional insights into the antibacterial mechanism of NIG, scanning electron microscopy (SEM) and transmission electron microscopy (TEM) were performed. Ampicillin, a β-lactam antibiotic, was used as a positive control since it blocks the synthesis of peptidoglycan, thus resulting in cell bursting due to osmotic pressure. Similar to the effects of ampicillin, collapse and lysis of bacteria were observed in the NIG-treated cells (4 × MIC, 6 h) *via* SEM (**Figure 4A**, **Figure S3**). Through TEM, we also found a ruptured cell wall and membrane, as well as leakage of intracellular material, upon NIG treatment (**Figure 4A**, **Figure S3**). Propidium iodide staining of the strain incubated with NIG showed red fluorescence, while no fluorescence was found in the control group (**Figure 4B**), supporting the hypothesis that NIG increases membrane permeability of *S. aureus*. Thus, NIG disrupted the membrane structure and permeability barrier.

### NIG disrupts ATP production and electron transport chain on bacterial membranes

As an ionophore, NIG may disturb proton motive force (PMF) across the cellular membrane, the electrical and chemical potential across the cellular membrane (Mitchell, 1966). Cellular change in electric potential (ΔΨ) and transmembrane proton gradient (ΔpH) are two key factors of PMF (Chen and Lo, 2016). Therefore, we then characterized the change in ΔΨ and ΔpH after NIG treatment. Daptomycin (DAP), a lipopeptide antibiotic, dissipated ΔΨ and served as the positive control (Le et al., 2020). In contrast, an increase in ΔΨ was observed after incubation with NIG (**Figure S4A**), which is consistent with previous research findings (Stokes et al., 2020). And the increase in ΔΨ appeared to be NIG concentration-independent (**Figure S4B**). The increase in ΔΨ induced by NIG may be due to the dissipation of ΔpH since ΔΨ and ΔpH are interdependent. A shift in extracellular pH to an acidic value will cause an increase in ΔpH across the membrane (Booth, 1985). The MIC of NIG decreased the most as the extracellular pH shifted from slightly basic to slightly acidic conditions while further decrease or increase in pH values conferred little change in NIG MIC (**Figure 4C**). The pH effect on charge of NIG was less likely responsible the MIC changes observed because when pH increased from 5.5 to 6.5, the NIG charge ([A^-^]%) increased 6% without affecting MIC while when pH increased from 6.5 to 7.5 and higher values, NIG charge changed little but its MIC increased by 2-8-fold (**Supplementary Table S4**). These data suggest that NIG might dissipate ΔpH mostly at near neutral pH, making shift of extracellular pH from neutral to either direction to affect NIG MIC the most.

Since PMF is essential for the production of ATP by F_0_F_1_-ATPase, dissipation of this electrochemical gradient might result in the cessation of ATP production (Farha et al., 2013). As shown in **Figure 4D**, upon NIG addition, there was a dramatic decrease in intracellular ATP levels to 25% of that of the control. Furthermore, the dissipation of PMF by NIG treatment might ultimately influence the electron transport chain (ETC) because of the interplay between PMF and ETC (Farha et al., 2013). Indeed, when treated with 1 × MIC of NIG, the reduction of iodonitrotetrazolium chloride (INT) to its formazan product by the ETC (Altman, 1976) decreased 50%, indicating that electron transport across the cytoplasmic membrane was impaired upon NIG treatment (**Figure 4E**). Collectively, these results revealed that NIG disrupts PMF and depletes the energy supply.

### No-cross-resistance to NIG and other representative anti-Gram-positive bacteria antibiotics

**Figure 5:**
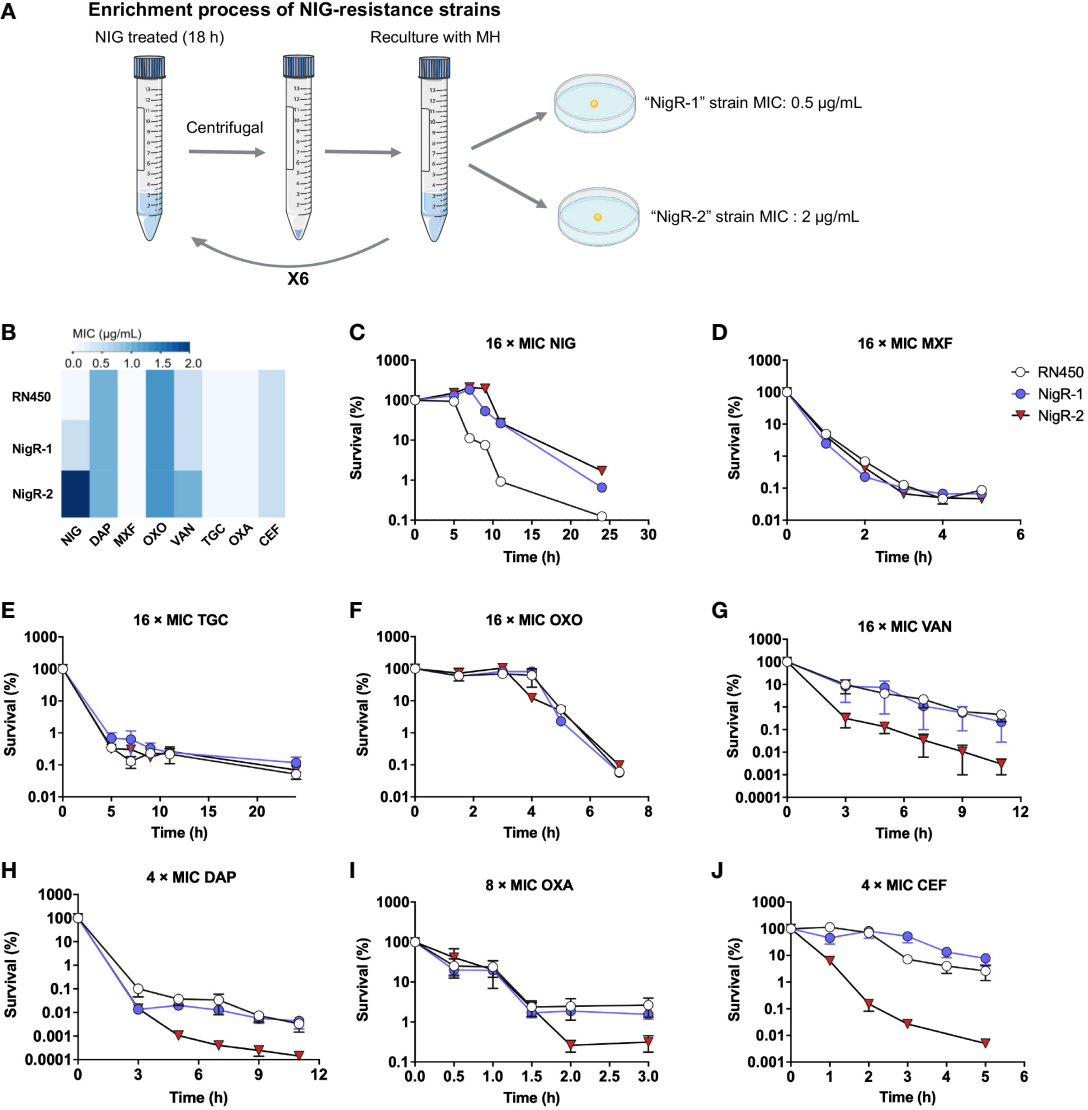
Lack of cross-resistance to other antimicrobials by *S. aureus* mutants acquired “resistance” to nigericin. **(A)** Schematic representation of enrichment used to obtain nigericin-resistant mutants of *S. aureus* strain RN450. **(B)** Comparison of MIC of various classes of antibiotics against RN450 and its nigericin-resistance mutant strains. **(C–J)** Effect of nigericin resistance mutations on killing by nigericin (NIG, **C**), moxifloxacin (MOX, **D**), tigecycline (TGC, **E**), oxolinic acid (OXO, **F**), vancomycin (VAN, **G**), daptomycin (DAP, **H**), oxacillin (OXA, **I**), and cefoxitin (CEF, **J**) for the indicated times and concentrations. Data are average of 3 biological replicates; error bars indicate SD.

Studying isolated NIG-resistant strains would help further elucidate the bactericidal mechanism of NIG. However, the previous serial passaging of *S. aureus* RN450 cultures, challenged with NIG, failed to produce NIG-resistant mutants (**Figure 2D**). Moreover, we were unable to obtain spontaneous NIG-resistant mutants by plating 10^9^ colony-forming units (CFU) of *S. aureus* RN450 on agar containing various concentrations (up to 32 × MIC) of NIG. Therefore, we performed another type of enrichment by treating *S. aureus* RN450 with 16 × MIC NIG for 18 h, washing the bacteria with saline (0.9% NaCl) three times, and culturing the surviving bacteria in drug-free medium. This procedure was repeated six times. Then single colonies were grown on drug-free agar, picked, and tested for MIC. Isolates that maintained MIC values 4-fold or greater than parental RN450 after recovery culturing for five daily passages were considered NIG-nonsusceptible (**Figure 5A**, **Supplementary Table S5**). Finally, two NIG-resistant strains were obtained, termed “NigR-1” and “NigR-2”, with a 4-fold and a 16-fold elevation in MIC, respectively (**Figure 5B**, **Supplementary Table S5**). Time-killing curves were determined for the NIG-resistant strains with NIG treatment. Compared with that on the wild-type *S. aureus* RN450, there was a more than 10-fold decrease in the bactericidal effect of NIG on NigR-1 and NigR-2 (**Figure 5C**). According to the characteristic drug responses of resistance, tolerance, and persistence (Brauner et al., 2016), it seemed that NigR-1 and NigR-2 have both resistance and tolerance characteristics for NIG.

We then tested the susceptibility of NigR-1 and NigR-2 on a training set of anti-Gram-positive bacteria antibiotics that represent seven distinct MoAs. The MIC values of MXF, TGC, oxolinic acid (OXO), VAN, DAP, OXA and cefoxitin sodium (CEF) were almost the same among the wild type, NigR-1, and NigR-2 (**Figure 5B**). Moreover, in contrast to that of NIG (**Figure 5B**), the time-killing curves of these 7 antibiotics also indicated that they retained bactericidal activity against the two NIG resistant mutants (**Figures 5C–J**). These results are consistent with NIG being effective against a variety of clinical Gram-positive MDR bacteria (**Figure 1B**). No-cross-resistance was demonstrated between NIG and these tested antibiotics. Thus, NIG may pose a MoA distinct from those of known classes of anti-Gram-positive bacteria antibiotics.

### Involvement of the GraSR two-component regulatory system in NIG action

Surprisingly, NigR-2 exhibited a phenotype that was less susceptible to NIG but more susceptible to VAN, DAP, OXA and CEF than NigR-1 (**Figures 5G–J**). We wondered whether these sensitivity differences could be attributed to the genetic alterations of the strains. Therefore, we performed next-generation sequencing on NigR-1 and NigR-2. The results showed that they both carried a frameshift mutation in the *merR* gene encoding a MerR family HTH-type transcriptional regulator that activates the expression of a multidrug resistance efflux pump (Liao and Sauer, 2012) (**Supplementary Table S6**). However, efflux pump should have little effect on agents that target cell membrane from outside of bacterial cells, such as nigericin. Even if efflux somehow has an effect on nigericin susceptibility, loss of function of MerR is expected to reduce the expression of the efflux pump, which would have decreased rather than increased nigericin MIC. Thus, the frameshift mutation in *merR* cannot be accounted for the increase in MIC with the two mutant strains.

An additional frameshift mutation in the *graS* gene was identified in NigR-2 (**Supplementary Table S6**). The histidine kinase GraS and the response regulator protein GraR compose the GraSR two-component system, which senses extracellular stimuli and transduces signals to activate the downstream *mprF* and *dltABCD* operons. Products encoded by *mprF* and *dltABCD* mediate an increase in positive cell surface charge through D-alanylation of wall teichoic acid, and lysinylation of phosphatidyl-glycerol within the cell membrane (Meehl et al., 2007; Muzamal et al., 2014). Such positive charge is expected to repel wall/membrane-targeting agents that are also positively charged.

**Figure 6:**
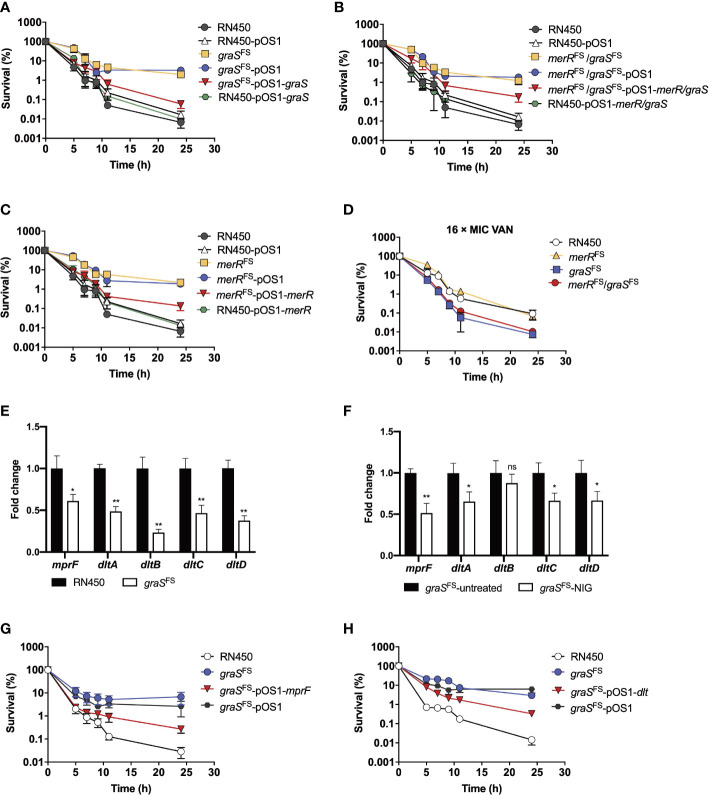
Involvement of the GraSR two-component regulatory system in reduced nigericin activity against of *S. aureus*. **(A–C)** Viability of *S. aureus* RN450 strains having deficiencies plus or minus complementary expression of *graS, merR*, or both upon treatment with nigericin (16 × MIC). *merR* ^FS^ = *merR* with a Thr184 frameshift; *graS* ^FS^ = *graS* with an Asn147 frameshift. pOS1 is a plasmid vector used for complementary expression of gene in *S. aureus*. **(D)** Increased killing by vancomycin (VAN) of *S. aureus* RN450 *graS* mutant strains. **(E)** Change in *dlt* and *mprF* mRNA expression levels in *graS* ^FS^ and *S. aureus* RN450. Levels of each mRNA in *graS* ^FS^ were normalized to those of corresponding mRNA in *S. aureus* RN450. Data are average of 3 biological replicates; error bars indicate SD. *p < 0.1; **p < 0.01. **(F)** Change in *dlt* and *mprF* mRNA expression levels in *graS* ^FS^ strain treated or untreated with NIG. The *graS* ^FS^ strain was treated with 2 × MIC nigericin (NIG) for 1 h. mRNA level of each gene was normalized to untreated control. Data are average of 3 biological replicates; error bars indicate SD. ns, not significant; *p < 0.1; **p < 0.01. **(G)** Partial reversal of *graS* deficiency-mediated protection from nigericin killing by ectopic expression of *mprF*. *S. aureus* R450 strains were treated with 16 × MIC of nigericin for the indicated times before samples were taken and processed for viability. **(H)** Partial reversal of *graS* deficiency-mediated protection from nigericin killing by ectopic expression of *dlt*. *S. aureus* RN450 strains were treated with 16 × MIC of nigericin for the indicated times before samples were taken and processed for viability. **(D, G, H)** Gene designations are as in panels **A-C**. Data in panels **(A–D, G, H)** are average of 3 biological replicates; error bars indicate SD.

To determine whether the distinct *graS* expression accounted for the susceptibility differences between NigR-1 and NigR-2, we used an inducible counterselection technology for targeted gene editing in *S. aureus* (Bae and Schneewind, 2006). Although the *graS* ^FS^ strain (*graS* with an Asn147 frameshift) showed an MIC of 0.125 μg/mL, the same as that of the wild-type RN450 (**Supplementary Table S7**), it was more tolerant to NIG killing since the survival rate of strains harboring *graS* ^FS^ mutations was 100-1000-fold higher than that of RN450. The *graS* ^FS^-pOS1-*graS* strain that expressed complementary wild-type *graS* in the *graS* ^FS^ mutant strain showed partially restored sensitivity to NIG. However, overexpression of *graS* ^FS^ in wild-type RN450 did not increase the sensitivity to NIG (**Figure 6A**). Thus, a loss, not a gain of function by *graS* accounted for the reduced NIG activity. The MIC for the *merR* ^FS^/*graS* ^FS^ double mutation strain (strain NigR-2) showed a 4-fold increase in MIC compared with that for the *merR* ^FS^ single mutant (**Supplementary Table S7**). The *merR* ^FS^/*graS* ^FS^ double mutation showed an increased survival (100-1000-fold higher) upon NIG treatment. Replenishing wild-type *merR* and *graS* resentitized the *merR* ^FS^/*graS* ^FS^ double mutation strain to NIG (**Figure 6B**). Similar results were also observed in *merR* ^FS^-related strains (**Figure 6C**). In addition, collapse and lysis of the strains harboring *merR* ^FS^ or *graS* ^FS^ mutations were not observed upon NIG treatment (**Supplementary Figure S5**). Similar to the result of NIG-resistance strains (**Figure 5G**), the *merR* ^FS^/*graS* ^FS^ mutation strains exhibited a phenotype that was more sensitive to VAN than *merR* ^FS^ mutation strain (**Figure 6D**).

The *mprF* and *dltABCD* operons are downstream of the GraSR two-component system. We next determined their expression levels by real-time PCR. As shown in **Figure 6E**, the mRNA levels of the *dlt* and *mprF* operon in *graS* ^FS^ mutant strain were significantly reduced compared to those of the wild-type strain (RN450). Upon NIG treatment, the mRNA levels of the *dlt* and *mprF* operon in *graS* ^FS^ mutant strain further decreased as compared to those of untreated samples. (**Figure 6F**). Overexpression of *mprF* and *dlt* gene re-sensitized the resistant strain carrying the *graS* frameshift mutation to NIG, as the survival rates decreased 100-fold (**Figures 6G, H**). Therefore, the decrease in the positive charge on the cell membrane by depletion of the GraSR two-component regulatory system leads to NIG resistance and tolerance. Collectively, these data indicate that frameshift mutations in both the *merR* and *graS* genes contribute to tolerance and resistance to NIG, but the additional mutation in *graS* leads to increased sensitivity to VAN, DAP, OXA and CEF, likely through reduction in positive charge in the cell wall and membrane.

## Discussion

Despite the fact that multidrug resistance in bacterial pathogens poses a significant clinical threat to human health, pharmaceutical research and development have failed to meet the urgent need for new antibiotics. Here, we reveal that NIG is a potent antibacterial candidate against various MDR Gram-positive bacteria, especially MRSA and VRE, which are ranked the highest in the WHO priority antimicrobial resistance pathogen list. Of note, NIG eliminated challenging persisters as well as established biofilms, which are intractable problems that could result in infection recurrence and treatment failure. Even VAN, the last line of defense against Gram-positive bacterial infections, has no effect on persisters and biofilms. We were also unable to obtain NIG-resistant mutants either in an agar-plated assay or with more than 32 days of serial passaging in culture, indicating NIG’s low propensity for resistance development. Moreover, NIG exhibits considerable *in vivo* efficacy in deep-seated mouse biofilm infection model, a model of chronic trauma infection, and a murine model of acute bloodstream infection. Taken together, these favorable features of NIG make it a promising anti-MDR strain antibiotic candidate for further investigation in clinical use.

Since persisters can survive through growth arrest during lethal antibiotic exposure, antibiotics that inhibit bacterial cell wall synthesis, protein synthesis, or nucleic acid replication are ineffective against persisters. Therefore, the bacterial membrane bilayer has been proven to be one of the most direct targets to kill persisters (Gollan et al., 2019). As a polyether ionophore, NIG has been shown to form coordination complexes with K^+^ and transport K^+^ across the lipid bilayer, leading to a decrease in the intracellular K^+^ concentration and an increase in the H^+^ concentration (Pressman et al., 1967). NIG increased propidium iodide uptake with *S. aureus*, indicating increased membrane permeability. That could explain synergistic lethal effects seen with the combination of TGC and NIG. Additional experiments showed that NIG disrupts bacterial membrane ATP production and ETC in *S. aureus*. That observation fits with NIG increasing membrane permeability and rupturing the cell membrane. Moreover, leakage of cytoplasmic material was readily observed by electron microscopy, confirming a previous study (Kumarsahu et al., 2020). Thus, the lethal action of NIG is likely due to membrane permeabilization. This distinct mode of action is expected to render NIG insensitive to factors that make Gram-positive bacteria resistant to existing antimicrobials.

In addition to the mutation in MerR, which functions as a multidrug transport activator, we also discovered a frameshift mutation in the GraS component of the GraSR two-component system. The GraSR system has been reported to greatly affect the resistance of *S. aureus* toward cationic antimicrobial peptides (CAMPs), VAN and DAP by increasing the net bacterial surface positive charge, causing electrostatic repulsion (Meehl et al., 2007; Neoh et al., 2008; Yang et al., 2012). We also found that the NIG-resistant strain NigR-2 harboring the *merR* ^FS^ */graS* ^FS^ double mutation was more sensitive to VAN, DAP, OXA and CEF than NigR-1 that has only the *merR* ^FS^ mutation (**Figures 5G–J**), indicating the important role of the GraSR system in antibiotic resistance. Further investigation using gene editing in *S. aureus* supported that depletion of GraS leads to NIG resistance, while activation of the GraSR system sensitizes bacteria to NIG. Due to this distinct MoA, cross-resistance to members of several antimicrobial classes (Quinolones, cyclo-lipopeptides, glycopeptides, tetracyclines, penicillins, and cephalosporins) was absent in NIG. This characteristic of NIG might be of great help for use in antibiotic combination or sequential treatment.

Overall, NIG demonstrates outstanding activity against drug-resistant, Gram-positive bacteria. A major barrier to further development is toxicity, which is seen with mammalian cells (Kawada et al., 1992), animal models (Gumila et al., 1997), and human studies (Pressman and Fahim, 1982). Members of the ionophore group differ in their cytotoxicity profiles, which encourages efforts to focus on those having low toxicity (Lin et al., 2021). The present work forms a baseline for evaluating anti-bacterial activity as those efforts advance. Particularly encouraging is the creation of a fluorinated derivative of NIG that lowers toxicity by 10-fold, while improving activity against Gram-negative bacteria (Kumarsahu et al., 2020). We expect such work could lead to the development of safe, effective ionophore antimicrobials.

## Data availability statement

The original contributions presented in the study are included in the article/**Supplementary Material**. Further inquiries can be directed to the corresponding authors.

## Ethics statement

The animal study was reviewed and approved by Laboratory Animal Center of Xiamen University.

## Author contributions

XD, XZ, LL, and DH conceived the project. XZhu, AH and GH performed the biological experiments, acquired and analyzed data. XZhu, AH, WW and HL evaluated drug efficacy *in vivo*. XS and DH collected and analyzed clinical strains. XD, XZ, LL, DH, QX, ZH, ZW and XW contributed to analysis and interpretation of biological data. XZhu, AH, LL, XZ and XD wrote the manuscript with comments from all authors. All authors read and approved the final manuscript. All authors contributed to the article and approved the submitted version.
