# Supplementary material for: Nigericin is effective against multidrug resistant gram-positive bacteria, persisters, and biofilms
Source: Front Cell Infect Microbiol. 2022 Dec 20;12:1055929. doi: 10.3389/fcimb.2022.1055929 (PMC9807916; doi:10.3389/fcimb.2022.1055929)
Supplement: Supplementary file 1 [file DataSheet_1.doc]

**SUPPLEMENTARY MATERIALS**

**Nigericin is effective against multidrug resistant Gram-positive bacteria, persisters and biofilms**

Xiaoli Zhu^1,2,5^, Anjin Hong^1,2,5^, Xihuan Sun^3,5^, Weijie Wang^4,5^, Guanghui He^1,2^, Huan Luo^4^, Zhenhua Wu^1,2^, Qingyan Xu^1,2^, Zhiyu Hu^1,2^, Xiaobing Wu^1,2^, Donghong Huang^3^,*, Li Li^1,2^,*, Xilin Zhao^4^,*, Xianming Deng^1,2^,*

**Table of Contents**

Supplementary Table S1. Activity of antimicrobials against pathogenic bacteria.

Supplementary Table S2. Detailed information for the clinically drug-resistant strains.

Supplementary Table S3. A synergy screening of nigericin in combination with representative antibiotics in *S. aureus* RN450.

Supplementary Table S4. NIG charge upon pH value change along with corresponding NIG MIC change.

Supplementary Table S5. Nigericin MIC of resistant isolates after passage in drug-free medium.

**S**upplementary Table S6. Mutations found in the open reading frames of nigericin-resistant strains of *S. aureus* R450.

Supplementary Table S7. Nigericin MIC with gene-edited mutants, corresponding complemented and overexpression strains.

Supplementary Figure S1. Bactericidal activity of nigericin is time dependent but not concentration dependent.

Supplementary Figure S2. Absence of lethal synergy between nigercin and oxacillin or moxifloxacin.

Supplementary Figure S3. SEM and TEM of RN450 after treatment with 4 x MIC nigericin for 6 h.

Supplementary Figure S4. Nigericin decreases proton motive force.

Supplementary Figure S5. SEM and TEM of *merR*^FS^ and *graS*^FS^ mutants of *S. aureus* RN450.

**Supplementary Tables**

**Supplementary Table S1. Activity of antimicrobials against pathogenic bacteria.**

| **Organism** | **Strains** | **MIC (μg/mL)** | | | |
| --- | --- | --- | --- | --- | --- |
|  |  | **NIG** | **VAN** | **AMP** | **PEN** |
| *Staphylococcus aureus* | MSSA RN450 | 0.125 | 0.5 | 1 |  |
|  | MRSA ATCC 33591 | 0.125 | 0.5 |  |  |
|  | MRSA USA300 | 0.125 | 0.5 |  |  |
| *Staphylococcus epidermidis* | ATCC 12228 | 0.125 |  |  |  |
| *Enterococcus. faecalis* | ATCC 29212 | 0.032 |  |  |  |
| *Enterococcus faecium* | W4-65 (VRE) | 0.1 | ≥32 |  |  |
|  | 75 (VRE) | 0.1 | 75 |  |  |
|  | W4-67 (VRE) | 0.032 | ≥32 |  |  |
| *Streptococcus pneumoniae* | ATCC 49619 | 0.004 |  |  | 0.25 |
|  | 65-90, PRSP | 0.004 |  |  | 8 |
| *Escherichia coli* | BW25113 | ≥32 |  | 4 |  |
| *Klebsiella Pneumoniae* | 43816 | ≥32 |  | ＜0.75 |  |
| *Acinetobacter baumannii* | ATCC 17978 | ≥32 |  | 0.25 |  |

NIG: nigericin, VAN: vancomycin, AMP: ampicillin, PEN: penicillin

**Supplementary Table S2. Detailed information for the clinically drug-resistant bacterial strains.**

| **Species (no. of isolates tested)** | **Intermediate breakpoint (μg/mL)** | | **Antimicrobial** | | **MIC (μg/mL)** | | | | |  |
| --- | --- | --- | --- | --- | --- | --- | --- | --- | --- | --- |
|  |  |  |  |  | **Range** | **50%** | | **90%** | |  |
| *S. aureus* (47) | | —^a^ | Nigericin^b^ | 0.125-0.25 | | | 0.125 | | 0.25 | |
|  | | 2 | Oxacillin | 1 - >4 | | | >4 | | >4 | |
|  | | 1 | Ciprofloxacin | ≤0.25 - >8 | | | 0.5 | | >8 | |
|  | | 0.5 | Moxifloxacin | ≤0.25 - >8 | | | ≤0.25 | | >8 | |
|  | | 0.5 | Erythromycin | ≤0.25 - >8 | | | >4 | | >8 | |
|  | | 1 | Quinupstin/ Dafopristin | ≤0.25 - 0.5 | | | ≤0.5 | | 0.5 | |
|  | | 2 | Vancomycin | ≤0.5 - 2 | | | ≤1 | | 1 | |
|  | | — | Tigecycline^b^ | ≤0.12 - 0.25 | | | ≤0.12 | | 0.25 | |
|  | | 1 | Rifampin | ≤0.5 - >32 | | | ≤0.5 | | 4 | |
|  | | 4 | Gentamicin | ≤0.5 - >16 | | | ≤1 | | >16 | |
|  | | 1 | Levofloxacin | ≤0.12 - >8 | | | 2 | | >8 | |
|  | | 0.5 | clindamycin | ≤0.25 - >8 | | | >2 | | >8 | |
|  | | 4 | Linezolid | 1 - 2 | | | 2 | | 2 | |
|  | | 4 | Tetracycline | ≤0.5 - >16 | | | >8 | | >16 | |
|  | | 32 | Nitrofurantoin | ≤16 - 32 | | | ≤16 | | 32 | |
|  | | 2/38 | Complex trimethoprim | ≤0.5 - >320 | | | ≤1/19 | | 40 | |
|  | | — | Tobramycin^b^ | ≤2 - 16 | | | ≤2 | | >8 | |
|  | | 8 | Teicoplanin | ≤1 - 2 | | | ≤1 | | 2 | |
| *S. epidermidis* (32) | | — | Nigericin^b^ | 0.0625-0.125 | | | 0.125 | | 0.125 | |
|  | | 0.5 | Oxacillin | ≤0.25 - >4 | | | >4 | | >4 | |
|  | | 1 | Ciprofloxacin | ≤0.5 - >8 | | | 1 | | >4 | |
|  | | 0.5 | Moxifloxacin | ≤0.25 - >8 | | | ≤0.25 | | >8 | |
|  | | 0.5 | Erythromycin | ≤0.25 - >8 | | | >4 | | >8 | |
|  | | 1 | Quinupstin/ Dafopristin | ≤0.25 | | | ≤0.25 | | ≤0.25 | |
|  | | 4 | Vancomycin | ≤0.5 - 16 | | | 1 | | 2 | |
|  | | — | Tigecycline^b^ | ≤0.12 - 0.5 | | | ≤0.12 | | 0.5 | |
|  | | 1 | Rifampin | ≤0.5 - >32 | | | ≤0.5 | | >2 | |
|  | | 0.12 | Penicillin | >0.25 - >8 | | | >0.25 | | >0.5 | |
|  | | 4 | Gentamicin | ≤0.5 - >16 | | | 4 | | >8 | |
|  | | 0.5/— | Inducible clindamycin | ≤0.12 - >8 | | | ≤0.12 | | >8 | |
|  | | 0.5 | clindamycin | ≤0.25 - >8 | | | ≤0.5 | | >8 | |
|  | | 4 | Linezolid | ≤0.5 - >4 | | | 2 | | 4 | |
|  | | 4 | Tetracycline | ≤0.5 - >16 | | | 2 | | >8 | |
|  | | 32 | Nitrofurantoin | ≤16 - 32 | | | ≤16 | | 32 | |
|  | | 2/38 | Compound trimethoprim | ≤1/19 - >320 | | | 2/38 | | >4/76 | |
|  | | — | Tobramycin^b^ | ≤2 - >8 | | | ≤2 | | >8 | |
| *E. faecalis* (4) | | — | Nigericin^b^ | 0.032-0.0625 | | | 0.032 | | 0.0625 | |
|  | | 1 | Ciprofloxacin | ≤0.5 - >8 | | | >8 | | >8 | |
|  | | — | Moxifloxacin^b^ | ≤0.25 - >8 | | | 4 | | >8 | |
|  | | 1 | Quinupstin/ Dafopristin | 1 - 4 | | | 4 | | 4 | |
|  | | 4 | Vancomycin | 1 | | | 1 | | 1 | |
|  | | — | Tigecycline^b^ | ≤0.12 | | | ≤0.12 | | ≤0.12 | |
|  | | 8 | Penicillin | 1 - 8 | | | 2 | | 8 | |
|  | | 2 | Levofloxacin | 0.5 - >8 | | | >8 | | >8 | |
|  | | 2 | Linezolid | 1 - 2 | | | 2 | | 2 | |
|  | | 4 | Tetracycline | >16 | | | >16 | | >16 | |
|  | | 8 | Ampicillin | ≤2 | | | ≤2 | | ≤2 | |
| *S. pneumoniae* (25) | | — | Nigericin^b^ | 0.002-0.0625 | | | 0.004 | | 0.004 | |
|  | | 1 | Moxifloxacin | ≤0.25 - 0.25 | | | ≤0.25 | | ≤0.25 | |
|  | | 0.25 | Erythromycin | ≤0.0625 - >4 | | | >4 | | >4 | |
|  | | — | Vancomycin^b^ | ≤0.5 - ≤1 | | | ≤0.5 | | ≤0.5 | |
|  | | 0.06 | Penicillin | ≤0.06 - >8 | | | 4 | | >8 | |
|  | | 2 | Levofloxacin | ≤0.5 - 2 | | | 1 | | 2 | |
|  | | 0.25 | clindamycin | ≤0.03125 - >1 | | | >1 | | >1 | |
|  | | 2 | Linezolid | ≤1 - 1 | | | ≤1 | |  | |
|  | | 1 | Tetracycline | ≤0.5 - >16 | | | >8 | | >16 | |
|  | | — | Complex trimethoprim^b^ | ≤0.5/9.5 - 80 | | | >2/38 | | 40 | |
|  | | 4 | Chloramphenicol | ≤2 - 8 | | | ≤2 | | 4 | |
|  | | 2 | Amoxicillin | ≤0.06 - >8 | | | 4 | | >8 | |
|  | | 0.25 | Meropenem | ≤0.0625 - 1 | | | 0.25 | | 0.5 | |
|  | | 1 | Cefepime | ≤0.5 - >2 | | | 1 | | 1 | |
|  | | 1 | Cefotaxime | ≤0.06 - >2 | | | 1 | | >2 | |
| *S. agalactiae* (6) | | — | Nigericin^b^ | 0.0625 | | | 0.0625 | | 0.0625 | |
|  | | — | Moxifloxacin | ≤0.25 - 4 | | | ≤0.25 | | 4 | |
|  | | — | Quinupstin/ Dafopristin | ≤0.25 - 0.5 | | | ≤0.25 | | 0.5 | |
|  | | — | Vancomycin | ≤0.5 | | | ≤0.5 | | ≤0.5 | |
|  | | — | Tigecycline | ≤0.12 | | | ≤0.12 | | ≤0.12 | |
|  | | — | Penicillin | ≤0.12 | | | ≤0.12 | | ≤0.12 | |
|  | | — | Levofloxacin | 0.5 - >8 | | | 1 | | >8 | |
|  | | — | clindamycin | ≤0.25 - >8 | | | >8 | | >8 | |
|  | | — | Linezolid | ≤0.5 - 2 | | | 1 | | 2 | |
|  | | — | Tetracycline | ≤1 - >16 | | | >16 | | >16 | |
|  | | — | Ampicillin | ≤0.25 | | | ≤0.25 | | ≤0.25 | |

^a^not determined. ^b^There is currently no CLSI-recommended breakpoint.

**Supplementary Table S3. A synergy screening of nigericin in combination with representative antibiotics against *S. aureus* RN450.**

| **Antibiotic** | **MIC_A_ (μg/mL)** | **FIC_A_** | **MIC_NIG_ (μg/mL)** | **FIC_NIG_** | **FIC index** |
| --- | --- | --- | --- | --- | --- |
| Tigecycline | 0.125 | 0.125 | 0.125 | 0.25 | 0.375 |
| Linezolid | 0.25 | 0.125 | 0.125 | 0.5 | 0.625 |
| Oxacillin | 0.125 | 0.25 | 0.125 | 0.25 | 0.5 |
| Rifampicin | 0.002 | 0.125 | 0.125 | 0.5 | 0. 625 |
| Vancomycin | 1 | 0.5 | 0.125 | 0.125 | 0.625 |
| Daptomycin | 0.75 | 1 | 0.125 | 0.125 | 1.125 |
| Kanamycin | 1 | 0.125 | 0.125 | 0.5 | 0.625 |
| Moxifloxacin | 0.125 | 0.125 | 0.125 | 0.25 | 0.375 |

Fractional inhibitory concentration (FIC) indices were calculated against *S. aureus* RN450 using checkerboard assays with various antibiotics and nigericin. MIC_A_ is the minimum inhibitory concentration (MIC) of each antibiotic listed in the antibiotic column alone. MIC_NIG_ is the MIC of nigericin alone. FIC_A_ is the FIC of each antibiotic listed in the antibiotic column in the presence of NIG. FIC_NIG_ is the FIC of nigericin in the presence of each antibiotic listed in the leftist column.

**Supplementary Table S4.** **NIG charge upon pH value change along with corresponding NIG MIC change.**

| pH | [A^-^]%^a^ | MIC (μg/mL) | Fold change of MIC |
| --- | --- | --- | --- |
| 5.5 | 93.33 | 0.0625 |  |
| 6.5 | 99.30 | 0.0625 | 1-fold |
| 7.0 | 99.78 | 0.125 | 2-fold |
| 7.5 | 99.93 | 0.125 | 2-fold |
| 8.5 | 99.99 | 0.5 | 8-fold |
| 9.5 | 99.99 | 0.5 | 8-fold |

^a^pKa = -log_10_([H^+^]×[A^-^]/[HA]). The pKa of NIG was predicted to be 4.351 using Chemdraw. The percentage of [A^-^] was calculated by the equation [A^-^]/[HA] =10^ (pH- pKa).

**Supplementary Table S5. Nigericin MIC of resistant isolates after passage in drug-free medium for five generations.**

| **Strains** | NIG MIC (μg/mL) | | | | |
| --- | --- | --- | --- | --- | --- |
|  | Round 1 | Round 2 | Round 3 | Round 4 | Round 5 |
| RN450 | 0.125 | 0.125 | 0.125 | 0.125 | 0.125 |
| NigR-1 | 0.5 | 0.5 | 0.5 | 0.5 | 0.5 |
| NigR-2 | 2 | 2 | 2 | 2 | 2 |

**Supplementary Table S6. Mutations found in the open reading frames of nigericin-resistant strains of *S. aureus* R450.**

| **Mutant Strain** | **Mutated gene** | **Function** | **Base change** | **Amino acid change** |
| --- | --- | --- | --- | --- |
| NigR-1 | *merR* | MerR family HTH type transcriptional regulator | 214A>deletion | Thr184frame shift |
| NigR-2 | *merR* | MerR family HTH type transcriptional regulator | 214A>deletion | Thr184frame shift |
|  | *graS* | Two-component sensor histidine GraSR | 440A>deletion | Asn147frame shift |

**Supplementary Table S7. Nigericin MIC with gene-edited mutants, corresponding complemented and overexpression strains.**

| **Strains** | **NIG (μg/mL)** |
| --- | --- |
| RN450 | 0.125 |
| RN450-pOS1 | 0.125 |
| NigR-1 | 0.5 |
| *merR*^FS^ | 0.25 |
| *merR*^FS^-pOS1 | 0.25 |
| *merR*^FS^-pOS1-*merR* | 0.125 |
| RN450-pOS1-*merR* | 0.125 |
| *graS*^FS^ | 0.125 |
| *graS*^FS^-pOS1 | 0.125 |
| *graS*^FS^-pOS1-*graS* | 0.125 |
| RN450-pOS1-*graS* | 0.125 |
| NigR-2 | 2 |
| *merR*^FS^ */graS*^FS^ | 0.5 |
| *merR*^FS^ */graS*^FS^-pOS1 | 0.5 |
| *merR*^FS^ */graS*^FS^-pOS1-*merR/graS* | 0.125 |
| RN450-pOS1-*merR/graS* | 0.125 |
| *graS*^FS^-pOS1-*mprF* | 0.125 |
| *graS*^FS^-pOS1-*dlt* | 0.125 |

**Supplementary Figures**

**
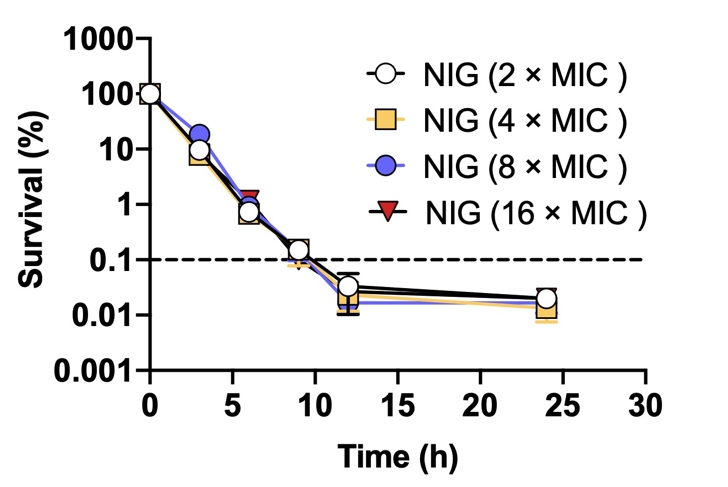
**

**Supplementary Figure S1. Bactericidal activity of nigericin is time dependent but not concentration dependent.** Survival of exponentially growing *S. aureus* RN450 after treatment with the indicated concentrations of nigericin. Individual data points are averages of 3 biologically independent experiments; error bars indicate standard deviation.


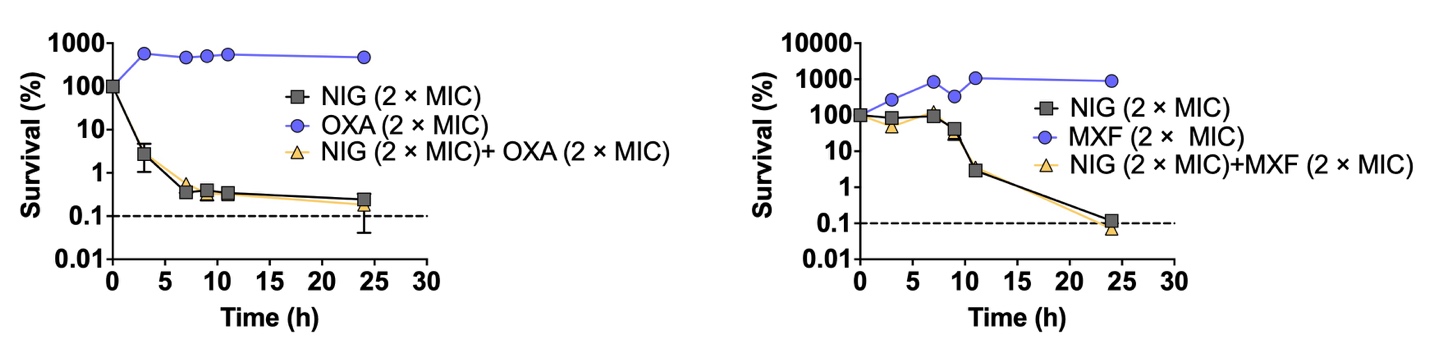


**Supplementary Figure S2. Absence of lethal synergy between nigericin and oxacillin or between nigericin and moxifloxacin.** Survival rate of *S. aureus* RN450 upon treatment of nigericin alone or in combination with oxacillin (OXA left) or moxifloxacin (MXF right). Individual data points are averages of 3 biologically independent experiments; error bars indicate standard deviation.

**
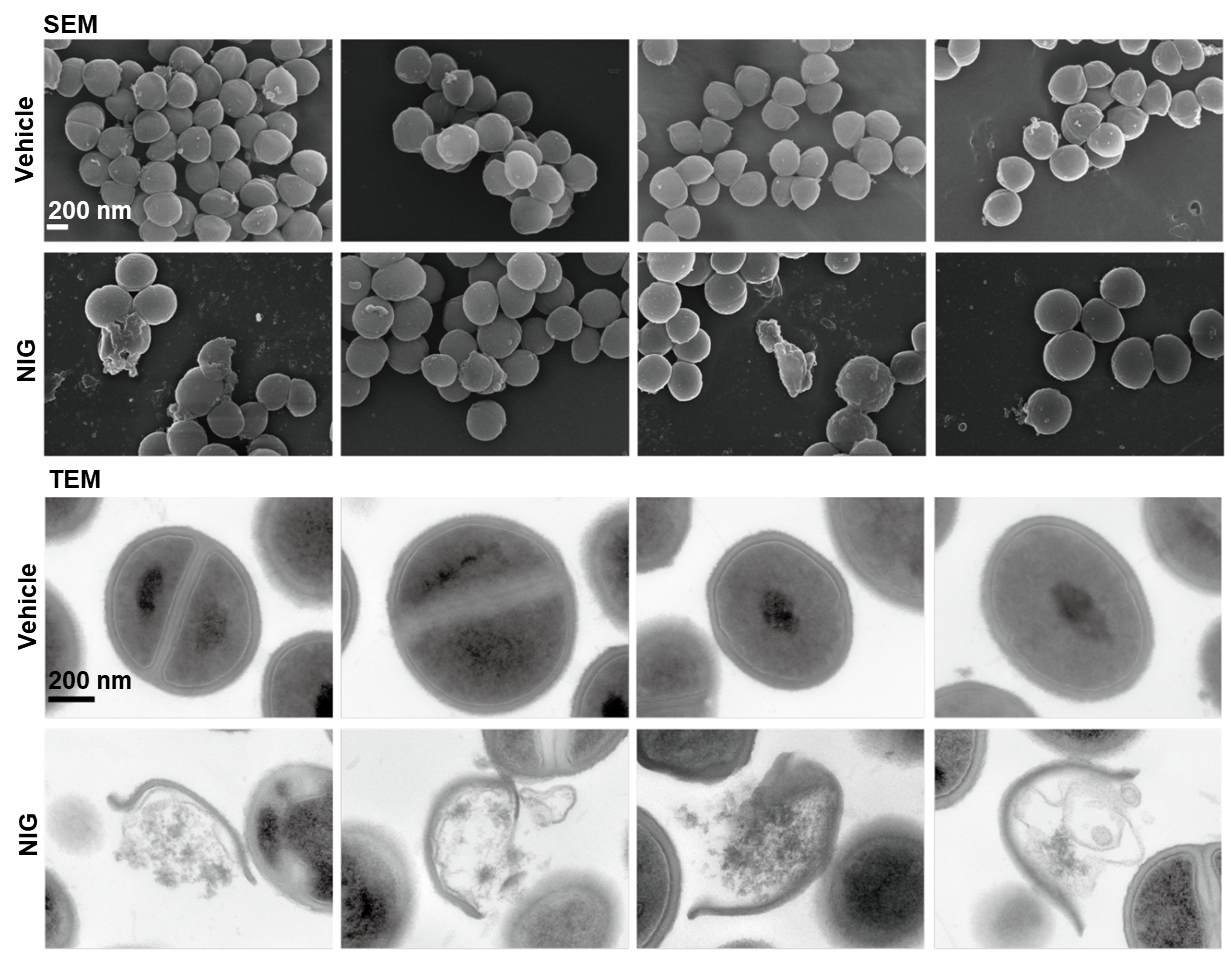
**

**Supplementary Figure S3. SEM and TEM of RN450 after treatment with 4 x MIC nigericin for 6 h**. Images are representative of 208 replicates from three independent bacterial preparations. Scale bar, 200 nm.


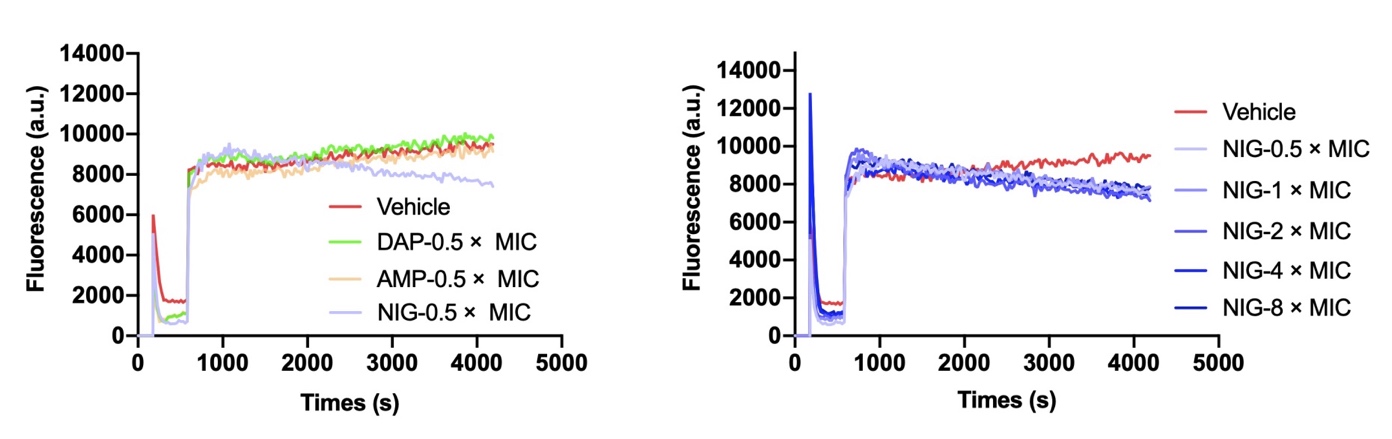


**A**

**B**

**Supplementary Figure S4. Nigericin decreases proton motive force.** (A) Fluorescence intensity changes of DiSC_3_(5), an agent that measures membrane potential, in *S. aureus* strain RN450 treated with daptomycin (DAP), ampicillin (AMP), or nigericin (NIG) at a concentration of 0.5 × MIC for 1 h. 0.1% EtOH was used as vehicle. (B) Fluorescence intensity changes of DiSC_3_(5) in *S. aureus* RN450 treated with the indicated concentrations of nigericin for 1 h.

**A**


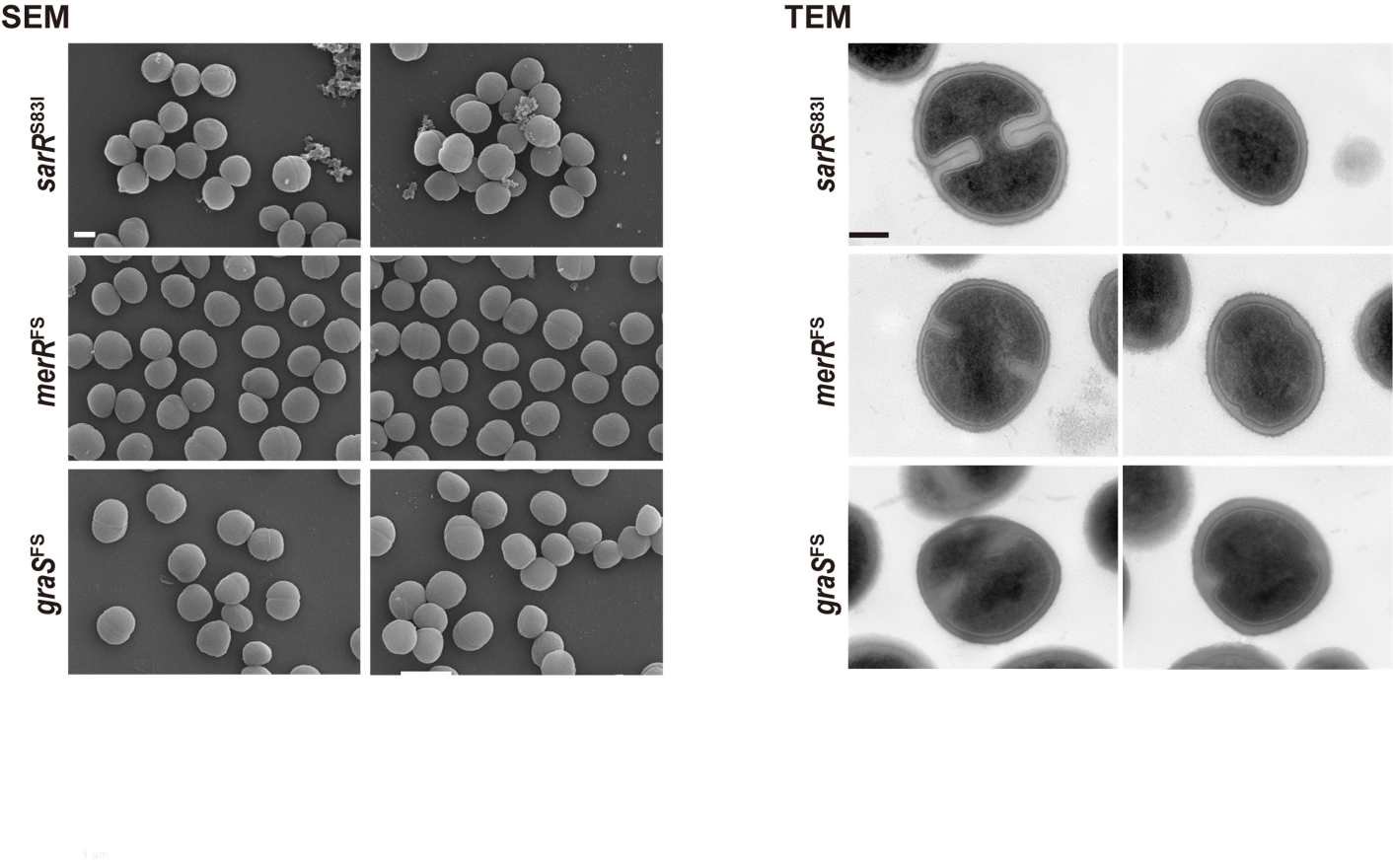


**B**

**SEM**

**TEM**

**Supplementary Figure S5. SEM and TEM of *merR*^FS^ and *graS*^FS^ mutants of *S. aureus* RN450.** SEM (A) and TEM (B) of *merR*^FS^ or *graS*^FS^ RN450 strains after treatment with 4 x MIC nigericin for 6 h. Scale bar, 200 nm.
